# Librarians' Electronic Resource Reviews Network (LERRN): a free citation database for resource reviews

**DOI:** 10.5195/jmla.2024.1862

**Published:** 2024-04-01

**Authors:** Louisa Verma

**Affiliations:** 1 lerrndb@gmail.com, Portland Cement Association Library Manager

**Keywords:** ERM, Electronic Resources, Reviews, Comparisons, Overviews, Databases

## Abstract

Electronic resource reviews written by librarians are a valuable way to identify potential content platforms and stay current on new resources. Resource-focused articles can also assist with learning about useful features, training others, and marketing to potential user groups. However, articles evaluating or highlighting innovative uses of resources may be published in disparate journals or online platforms and are not collocated. Small or solo-staffed libraries may not subscribe to library and information sciences databases or journals that contain reviews of electronic resources. And many of these reviews or other useful articles are open access. With this in mind, the main aim of the LERRN citation database was to create a freely available citation database that brings together electronic resource reviews and other content that can assist librarians in appraising and using electronic resources.

To build the database prototype, a list of journals was created from known journals, library association-related publications, Directory of Open Access Journals (DOAJ), and by searching on the JournalTOCsv (UK) website for library and information science-related titles. Citation and abstracts from journals containing content relevant to electronic resources are gathered into the free Zotero bibliography management tool through imported RSS feeds. Once articles are selected and tagged, they are imported into the Librarika online catalog (a low-cost, cloud-based integrated library system (ILS)) by a comma-separated values (CSV) template provided by the vendor. The LERRN database and website was created and is currently maintained, updated, and subsidized solely by the author.

The current database includes citations back to 2019 and focuses on electronic resources in the areas of science, technology, engineering, mathematics, and medicine. The author reviews selected journals' Zotero RSS feeds every other month to select citations that will go into the LERRN database. Once selected relevant citations are given four tag types in Zotero (year, resource name, topic, article type) to assist with browsing by Tags, Categories, Series, and Authors in the Librarika ILS. Articles are selected for inclusion based on their value in assisting with electronic resource purchasing, training, marketing, or use. In addition to reviews, article types also include overviews, comparisons, projects, search tips, book reviews, and general electronic resource management (ERM) articles. The database is updated six times a year in January, March, May, July, September, and November. A website was created on Google Sites to highlight the rationale, scope, and update frequency of the database. A list of journals indexed is also available on the website or under the Librarika series link. The What's New blog from the LERRN website can be subscribed to for update announcements.

**Figure 1:**
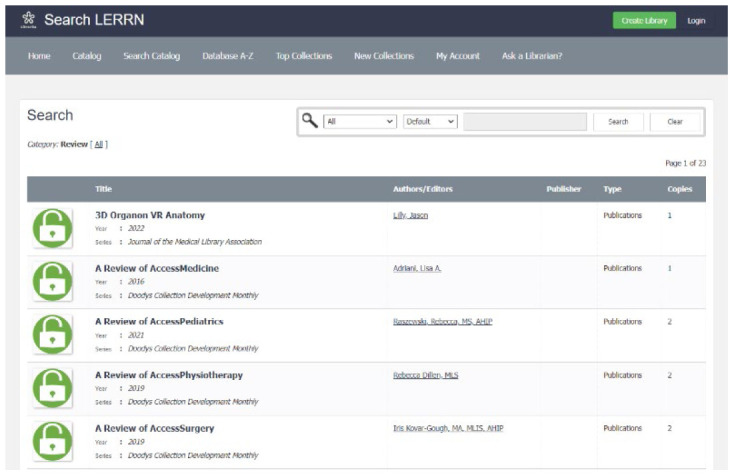
Librarians' Electronic Resource Reviews Network (LERRN).

The LERRN database, implemented in 2022, is in its early stages of development. Limitations include the use of the Librarika platform as an index & abstract database. Librarika is intended as an online catalog and, as such, it provides less flexibility for tailoring searching, browsing, or highlighting content but offers importing citations, automatic browsing, and linking to full text. Future development plans include expansion to include reviews for the social sciences and humanities and adding reviews further back than 2019 for resources that do not have a more recent review. To date, the database has over 8,000 page views and includes 920 articles (a majority published from 2019 to the present). It is the author's expectation that as content continues to be added to the database, it will become a useful tool for librarians.

Contact the author for more information.

